# Complex Housing, but Not Maternal Deprivation Affects Motivation to Liberate a Trapped Cage-Mate in an Operant Rat Task

**DOI:** 10.3389/fnbeh.2021.698501

**Published:** 2021-08-26

**Authors:** Aikaterini Kalamari, Jiska Kentrop, Chiara Hinna Danesi, Evelien A. M. Graat, Marinus H. van IJzendoorn, Marian J. Bakermans-Kranenburg, Marian Joëls, Rixt van der Veen

**Affiliations:** ^1^Department of Translational Neuroscience, UMC Utrecht Brain Center, University Medical Center Utrecht, Utrecht University, Utrecht, Netherlands; ^2^Department of Psychology, Education and Child Studies, Erasmus University Rotterdam, Rotterdam, Netherlands; ^3^Primary Care Unit, School of Clinical Medicine, University of Cambridge, Cambridge, United Kingdom; ^4^Faculty of Behavioral and Movement Sciences, Vrije Universiteit Amsterdam, Amsterdam, Netherlands; ^5^University Medical Center Groningen, Groningen University, Groningen, Netherlands; ^6^Brain Plasticity group, SILS Center for Neuroscience, University of Amsterdam, Amsterdam, Netherlands

**Keywords:** rats (all MESH terms), complex housing, maternal deprivation model, pro-social decision making, operant liberation task, social development, pro-social behavior

## Abstract

Early life environment influences the development of various aspects of social behavior, particularly during sensitive developmental periods. We studied how challenges in the early postnatal period or (early) adolescence affect pro-social behavior. To this end, we designed a lever-operated liberation task, to be able to measure motivation to liberate a trapped conspecific (by progressively increasing required lever pressing for door-opening). Liberation of the trapped rat resulted either in social contact or in liberation into a separate compartment. Additionally, a condition was tested in which both rats could freely move in two separate compartments and lever pressing resulted in social contact. When partners were not trapped, rats were more motivated to press the lever for opening the door than in either of the trapped configurations. Contrary to our expectations, the trapped configuration resulted in a *reduced* motivation to act. Early postnatal stress (24 h maternal deprivation on postnatal day 3) did not affect behavior in the liberation task. However, rearing rats from early adolescence onwards in complex housing conditions (Marlau cages) reduced the motivation to door opening, both in the trapped and freely moving conditions, while the motivation for a sucrose reward was not affected.

## Introduction

It has been demonstrated that social behavior can be affected by previous experiences, especially during sensitive developmental periods such as the early postnatal period and (early) adolescence (Marco et al., 2011; Sandi and Haller, 2015; Tzanoulinou and Sandi, 2016). In rodents, early life adversity in the first 2 weeks after birth-by depriving pups of maternal care or providing pups with poor quality of maternal care—can negatively affect social behavior, although this has not been as extensively studied compared to effects on cognition (Bonapersona et al., 2019). The maternal deprivation model has been previously shown to induce HPA-axis changes (Workel et al., 2001; Enthoven et al., 2010) and long-lasting structural (Loi et al., 2014; Sarabdjitsingh et al., 2017) and functional (Oomen et al., 2010, 2011; Derks et al., 2016; Loi et al., 2017) changes in the brain. Our lab has shown that this deprivation negatively affects adult behavioral inhibition and social discrimination (Kentrop et al., 2016, 2018). The other side of the coin is that favorable conditions during sensitive periods might benefit development and positively affect social behavior. For example, communal nesting, in which two or more mothers raise their pups together in a shared nest, was found to enrich the repertoire of social behaviors in mice (Branchi and Alleva, 2006; Branchi and Cirulli, 2014). Later in life, exposure to a more naturalistic and “enriched” environment, comprised of both social and physical enrichment and regular exposure to novelty increases brain plasticity and may positively influence the development of social skills (Würbel, 2001; Gubert and Hannan, 2019). Like in humans, the period of adolescence is a period in rodents in which brain circuitry implied in social behavior is still in development (Fuhrmann et al., 2015; Casey et al., 2019). Studies applying this “enrichment” during the adolescence period report reduced anxiety and enhanced learning, memory, and social behavior later on (van Praag et al., 2000; Simpson and Kelly, 2011; Crofton et al., 2015). We have recently found differential effects on social play in adolescence and social interest in adulthood in animals housed in enriched Marlau cages compared to standard housed animals (Kentrop et al., 2018). There is however not much known about the impact of early life conditions, both negative and positive, on pro-social behavior.

Pro-social behavior, i.e., behavior that benefits others, has been considered to benefit not only the well-being of the recipient but also the actor (Curry et al., 2018). It is built upon the three components of empathy, namely: (1) emotional contagion, i.e., the capacity to experience and share the emotions of others; (2) perspective taking, i.e., the ability to reason from another’s point of view; and (3) empathic concern, i.e., other-oriented emotions elicited by and congruent with the perceived welfare of someone in need (de Waal, 2008; Chen, 2018; Sivaselvachandran et al., 2018). Emotional contagion is regarded as an evolutionary well-preserved mechanism that helps individuals to survive, not only in dangerous savanna but also within social groups (Preston and de Waal, 2001; de Waal, 2008; Kim et al., 2019). Rodents, like many other animals, experience and learn from emotional contagion, as shown in studies on emotional contagion for pain (Church, 1959; Langford, 2006; Langford et al., 2010; Atsak et al., 2011; Pereira et al., 2012; Li et al., 2014; Cruz et al., 2020) and observational fear conditioning (Kavaliers et al., 2003; Jeon et al., 2010; Kim et al., 2010; Allsop et al., 2018; Keum and Shin, 2019; Nomura et al., 2019). Empathic concern and perspective taking, were initially thought to be characteristics unique to humans, but an increasing number of experimental studies challenge this idea and suggest that pro-social behavior and its underlying mechanisms can also be studied in non-human primates and other animals (De Waal and Preston, 2017; Pérez-Manrique and Gomila, 2018; Sivaselvachandran et al., 2018). Studies have shown that rats help distressed conspecifics in laboratory settings, e.g., pressing a lever to lower a rat that is suspended in the air (Rice and Gainer, 1962) and allowing a soaked rat to escape a pool of water (Sato et al., 2015). Bartal et al. (2011) developed a model in which a rat was trapped in a cylinder that could be manually opened by a conspecific. After being trained to operate the cylinder door, a high percentage of rats actively liberated trapped familiar conspecifics. Rats did not open the cylinder when it was empty or contained a toy rat, suggesting that the liberation act has reinforcing properties. In a follow-up study, rats were shown to liberate familiar but not unfamiliar conspecifics (Bartal et al., 2014), demonstrating that previous social experiences shape helping behavior later in life, in line with other studies (Langford et al., 2010; Burkett et al., 2016; Rogers-Carter et al., 2018).

We adapted this pro-social liberation model to an operant set-up where a rat is trapped in a cylinder blocked by automated doors. These doors can be opened by a free rat in an adjacent compartment, by means of lever pressing. To assess the motivation to liberate a trapped conspecific, the number of presses needed to open the doors is progressively increased over sessions, increasing the cost of liberation (in contrast to a single action). To distinguish between motivation to liberate a distressed conspecific and motivation for social contact (Silberberg et al., 2014), the task was performed using two configurations: one in which opening the door gave the trapped rat access to the cage of the liberator, enabling social contact; and one in which opening the door gave the trapped rat access to a separate compartment. Additionally, a third configuration was tested in which both rats could freely move in separate compartments and lever pressing resulted in gaining access to the other’s compartment. We also recorded ultrasonic vocalization (USV), an important communication channel for rats which might influence pro-social behavior. For adult rats, USVs can be divided into two categories that reflect different affective states: “alarm” calls in the range of 18–32 kHz (referred to as 22 kHz calls); and “appetitive” calls in the range of 33–96 kHz (referred to as 50 kHz calls; Portfors, 2007; Takahashi et al., 2010; Wright et al., 2010; Brudzynski, 2013; Wöhr and Schwarting, 2013). Calls in the 22 kHz domain are emitted during aversive situations such as direct danger, approaching danger, or emotional distress, whereas 50 kHz calls are usually emitted in pleasant situations and function to establish and maintain contact with conspecifics (Simola and Brudzynski, 2018; Brudzynski, 2019). Social enrichment can lead to an increase in ultrasonic communication and approach behavior in response to appetitive calls (Brenes et al., 2016).

To study the impact of both negative and positive early life environments on pro-social behavior in adulthood, we introduced two environmental manipulations: (1) manipulation of the early postnatal environment through 24 h maternal deprivation (MD) on postnatal day 3; and (2) manipulation of the adolescent environment through enriched complex housing (CH) from postnatal day 26 onwards, up and throughout testing in adulthood. All rats were tested in our automated operant version of the liberation task in adulthood.

## Materials and Methods

### Animals

**Figure 1:**
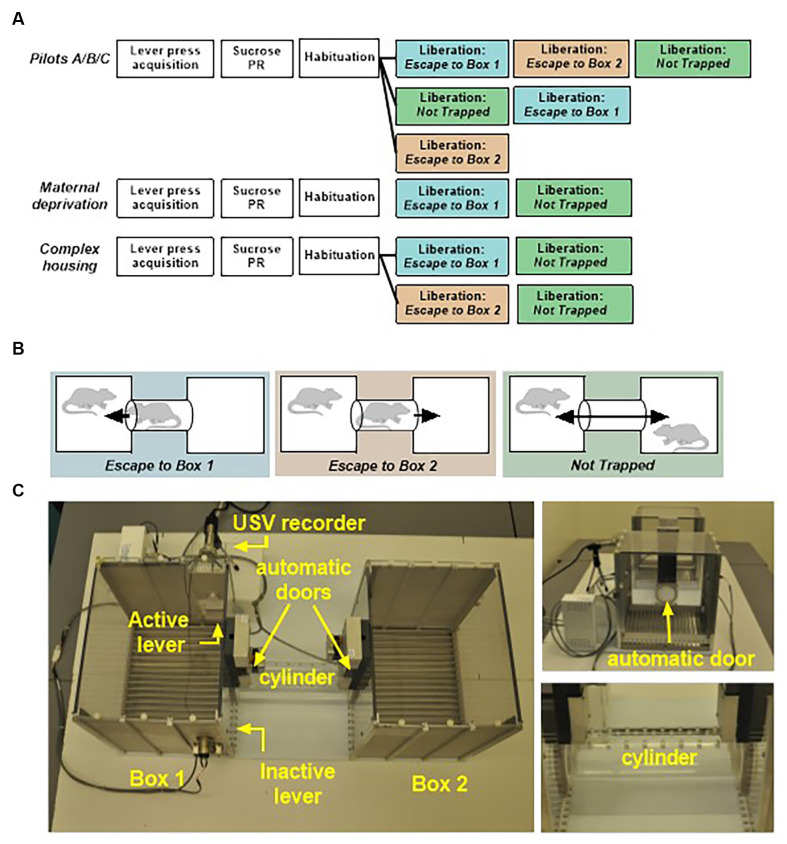
**(A)** Schematic overview of the behavioral protocols used in the pilots, the maternal deprivation experiment and the complex housing experiment. PR, progressive ratio. **(B)** Three different configurations in the liberation task were tested: (1) Escape To Box 1 in which the partner was trapped in the cylinder and could be liberated from the cylinder into box 1 (the test rat compartment), (2) Escape To Box 2 in which the partner was trapped in the cylinder and could be liberated into box 2, and (3) Not Trapped configuration in which the partner was situated in box 2 and lever presses of the test rat opened both doors. **(C)** Liberation task set-up: two operant chambers are connected through a removable cylinder. The cylinder can be closed on both sides by automated mechanical doors made of transparent Plexiglas with holes that allowed rats to see, smell and hear, but not touch each other.

Male and female Wistar breeding rats were obtained from Charles River Laboratories (Arbresle, France) at 8–10 weeks of age. All experimental rats were bred in-house. Only male rats were included in the experiments. Standard housed rats were kept in type IV Makrolon cages (37 × 20 × 18 cm) in temperature (21°C) and humidity (55%) controlled rooms with a 12 h light–dark cycle (lights on from 08:00–20:00) during breeding, and a reversed cycle (lights on from 20:00–08:00) from postnatal day 21 onwards. Unless stated otherwise, food and water were available ad libitum and all cages were provided with a woodblock as standard cage enrichment. Clean cages were provided and general health status was checked on a weekly basis. Animals were semi-randomly assigned to the experimental or control groups; with the constraint that a maximum of two rats from the same litter were assigned to the same group to minimize litter effects. Rats were tested in adulthood (90+ days of age) in pairs; one acting rat, referred to as *test rat*, and one stimulus rat, referred to as the *partner*. Rats participated either as test rat or partner. Where possible, siblings were housed together and became partners. All rats were previously tested in a pro-social two-choice task (Kentrop et al., 2020), with at least 2 weeks rest before entering the current experiment. The present study consists of a series of pilot experiments, an early life stress experiment with maternal deprivation (MD, *n* = 10 animals/experimental group); and a complex housing experiment (CH, *n* = 29 animals/experimental group), see Figure 1A for a schematic overview of all experiments. A total number of 18 pilot animals and 78 experimental animals was used in this study. Experiments were approved by the Central Authority for Scientific Procedures on Animals in the Netherlands (CCD project AVD115002016644). Animal care was conducted in accordance with the EC Council Directive of November 1986 (86/609/EEC).

### Breeding and Maternal Deprivation

Breeding started after the rats had been familiarized with our animal facility for at least 2 weeks. Two females were paired with 1 male for 10 days. After separation from the male, females stayed together for another week and were then individually housed to prepare for birth. Paper towels were provided to the mothers as nesting material. The day of birth was considered postnatal day 0. On postnatal day 3 the sex of the pups was determined and when necessary litters were culled to a maximum of 10 or supplemented to a minimum of 6 by adding pups from surplus animals from culled litters. Mothers and pups were placed back into their home cage within 2 min, except for litters in the MD experiment. During deprivation, the pups stayed together in their home cage (without the dam, which remained single housed in a separate cage without pups, with food and water ad libitum) and were transported to a different room. The cage with pups was placed on a heating pad (33°C) to prevent hypothermia; the pups were left undisturbed and they were not fed. After 24 h the cage was taken back to the original room and the mother was reunited with her litter. The week following deprivation, mothers from deprived litters were not provided with additional nesting material.

### Weaning and Complex Housing

Pups were weaned on postnatal day 21. Pups from the pilot experiments, MD experiment, and the standard housing condition in the housing experiment, were pair-housed in type IV Makrolon cages after weaning. Rats from the housing experiment assigned to the complex housing condition were housed in type IV Makrolon cages with three to four males from postnatal day 21 to 26 and then transferred to Marlau™ cages (Viewpoint, Lyon, France), housing 10 males per cage. Marlau cages are large, enriched cages (60 × 80 × 51 cm) that have 2 floors and provide a complex and challenging environment for the rats (Fares et al., 2013). The first floor contains a big compartment with three running wheels, a shelter, ad libitum access to water, two woodblocks, and a climbing ladder to the second floor, where a maze has to be passed to gain access to a tube leading to the food compartment on the first floor. *Via* a one-way passage rats can regain access to the bigger first-floor compartment. The maze was changed once per week (alternating between 12 different configurations), assuring novelty and sustained cognitive stimulation. Territorial dominance was avoided by the presence of two openings on each side of the maze. A more detailed description of the experimental setup is given elsewhere (van der Veen et al., 2015).

### Boldness Test

To determine which rats would become test rat and partner, rats were subjected to a boldness test. Boldness was measured by opening the lid of a cage halfway and measuring for each rat how much time was needed to rear and place at least one paw on the edge of the cage. This test was repeated three times on separate occasions and rats were ranked based on their cage emergence latency; the boldest rats (i.e., with the shortest emergence latency) were selected as test rats and the others as partners. The selection based on boldness was made to ensure that none of the test rats would have to be excluded from the experiment due to non-performance as a result of fear (replicated from the protocol by Bartal et al., 2011). For standard housed rats, the boldness test was conducted in the home cage and the selection was made within each pair. Complex housed rats were first removed from their complex home cage into type IV Makrolon transport cages (to which they were extensively habituated) and were left undisturbed for 30 min. After 30 min, rats were transferred one by one to an empty transport cage and tested individually. Rats with the highest boldness rank were selected as test rats.

### Acquisition of Lever Pressing and Progressive Ratio for Sucrose

#### Temporary Mild Food Restriction and Sucrose Pellet Habituation

In order to acquire lever pressing before the start of the liberation task, rats were trained with sucrose pellets in separate operant cages. The week before training, the rats received sucrose pellets in the home cage on three occasions to habituate to the taste of the pellets. Starting 1 week before the acquisition, animals were mildly food-restricted (3–4.5 g chow/100 g body weight) to attain 90–95% of their free-fed bodyweight (bodyweight loss monitored twice a week). Once the animals completed training and progressive ratio for sucrose (10 to 12 days), they were returned to an ad libitum feeding schedule and were left undisturbed for 5–7 days before they were habituated to the liberation apparatus.

#### Apparatus

Acquisition of lever pressing took place in separate operant chambers (30.5 × 24.1 × 21 cm, Med Associates, St. Albans, VT, USA) equipped with two retractable levers, a cue light above each lever, a house light (which illuminated the chamber with dim light) and a pellet magazine in between levers that provided rats with a sucrose pellet (45 mg, Formula P; Bio-Serv). These cages were of a different design and located in a different room compared to the liberation set-up. Rats could earn a sucrose pellet by pressing the active lever, presses on the inactive lever were registered but had no consequences. The operant chambers were enclosed in larger boxes equipped with exhaust fans that assured air renewal and masked background noises. Experimental contingencies were controlled and data were collected using MED-PC version 14.0 (Med Associates).

#### Behavioral Procedure

*Habituation*: Rats were habituated to the operant chambers in a 15 min session in which every minute the cue light above the active lever was turned on for 20 s in combination with the delivery of a sucrose pellet.

*Acquisition training*: A training session lasted 30 min and rats were trained twice a day (morning and afternoon). A session contained several trials, in each trial a sucrose pellet could be earned. Training started with a fixed ratio 1 (FR1) schedule of reinforcement where 1 active lever press was required for the delivery of a sucrose pellet. Initially, rats were trained on an FR1 protocol in which both levers were active (a sucrose pellet could be earned on each of the two levers) and trials were separated by a 10-s inter-trial-interval (ITI) until the acquisition criteria of 30 pellets within a session was reached. In the next training phase, one of the levers became the inactive lever on which no sucrose could be earned. Rats were subsequently trained on an FR1 (with an acquisition criteria of 30 pellets), FR3 (three lever presses were needed on the active lever to obtain a sucrose pellet, criteria of 30 pellets), and FR5 protocol (five lever presses were needed on the active lever to obtain a sucrose pellet, criteria of 18 pellets), with an ITI of 20 s. Sucrose pellet earning: Upon reaching the required number of presses to complete a fixed ratio, all levers retracted, the cue light above the active lever turned on to signal reward delivery and a sucrose pellet was dispensed in the pellet magazine.

*Progressive ratio*: To assess the motivation to work for a food reward, a progressive ratio (PR) protocol was conducted in which the number of required presses to earn a sucrose pellet progressively increased from 1–2, 4, 6, 9, 12, 15, 20, 25, 32, 40, 50, 62, 77, 95, 118, 145, 178, 219, 268, 328, etc. according to Richardson and Roberts (1996). A PR session lasted maximally 90 min or ended as soon as a rat did not reach the new ratio within 15 min of the previous one. PR was conducted twice, on two separate days. After the last PR test, rats returned to ad libitum feeding and were allowed to recover for at least 1 week before behavioral testing in the liberation task. For all acquisition training and PR sessions, the total numbers of rewards and active and inactive lever presses were recorded.

### Liberation Task

#### Apparatus

The liberation task was different in size and set-up from the sugar conditioning cages and located in a different room. Liberation was conducted in a set-up consisting of two operant chambers (29.5 × 23.5 × 27.3 cm, Med Associates, St. Albans, VT, USA) placed 25 cm apart and connected by a removable transparent Plexiglas cylinder (25 cm in length and 7.5 cm in diameter) with holes that provided fresh air (6 mm in diameter) for rats that were trapped in the cylinder (Figure 1C). The cylinder could be closed on both sides by automated mechanical doors made of transparent Plexiglas with holes (6 mm in diameter) that allowed rats to see, smell, hear but not touch each other. Depending on the configuration tested, one or both doors were programmed to open in response to lever pressing by the test rat. Box 1, the test rat compartment (left side), contained two levers; one active lever with a cue light situated above it, and one inactive control lever. To record ultrasonic vocalizations a microphone was placed behind a piece of wire mesh integrated into the upper part of the wall in box 1. Experimental contingencies were controlled by MED-PC IV Version 4.2 software (Med Associates, St. Albans, VT, USA). During testing, the number of active and inactive lever presses, door openings, and door opening latencies were recorded. Experiments were conducted under red light conditions during the dark (active) phase.

#### Behavioral Procedure

*Habituation to box and levers*: The rats that performed as actors were habituated to the set-up for 20 min without levers and with full access to box 1, box 2, and the cylinder (i.e., both doors were open). Following this, on four consecutive days rats were placed in the setup for 20 min on an FR1 (2x) and FR3 (2x) protocol in which both levers were present, the cylinder doors were closed (the cylinder empty) and pressing the active lever resulted in door opening of both doors for 1 min, giving access to the cylinder and box 2. Rats coupled lever pressing in this cage to door opening, and active pressing quickly declined.

*Configurations in liberation task*: Three different configurations in the liberation task were tested (Figure 1B): (1) *Escape to Box 1* in which the partner was trapped in the cylinder and could be liberated from the cylinder into box 1 (the test rat compartment); (2) *Escape to Box 2* in which the partner was trapped in the cylinder and could be liberated into box 2; and (3) *Not Trapped* in which the partner was situated in box 2 and lever presses of the test rat opened both doors. In all configurations, test rats were placed in box 1 and could open the cylinder door(s) by lever pressing, thereby liberating their partner from the cylinder or gain access to each other.

**Figure 2:**
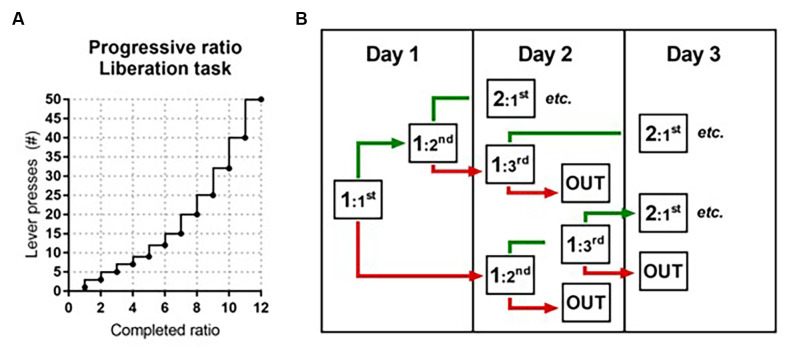
Schematic representation of the adapted progressive ratio protocol in the liberation task. **(A)** The progressive increase in the number of required lever presses to complete a ratio and open the cylinder door(s) (adapted from Richardson and Roberts, 1996). **(B)** Decision tree in the liberation task. If during the first attempt on a given day rats reached the required number of lever presses (ratio completed; in green), a second attempt followed. If during the first attempt on a given day rats failed to reach the required number of lever presses (ratio not completed; in red), rats were tested again the next day. Rats needed to complete a given ratio twice in maximally three attempts. When they failed to do this, the preceding ratio was recorded as the maximum ratio reached.

*(Adapted) Progressive Ratio*: To assess the motivation to liberate a trapped cage mate, a progressive ratio protocol was introduced. As a proxy for motivation, the required number of presses needed to liberate the cage mate per ratio progressively increased over sessions from 1–3, 5, 7, 9, 12, 15, 20, 25, 32, 40, 50, 62, 77, etc. (adapted from Richardson and Roberts, 1996) until rats reached a breakpoint. The breakpoint is the maximum amount of presses animals are willing to make within 10 min to liberate their partner. Thus, if a rat completed five ratios (five liberations) in the PR, this means that the maximum amount of presses to liberate the partner rat was nine and the sixth ratio was not completed (i.e., the rat did not reach the next 12 presses within 10 min, see Figure 2A). The difference with the PR for sucrose is that the ratios did not progress within a session, but between sessions. Rats were tested once a day in one or two consecutive sessions in which the test rat was placed in box 1 and could open the door(s) by reaching the required amount of lever presses within 10 min. As soon as rats completed a given ratio, the door(s) opened and the session ended 5 min later. If rats did not complete a ratio, the session ended after 10 min and both rats were taken out of the test. In order to progress, rats needed to complete a given ratio twice in maximally three sessions (Figure 2B).

### Recording and Analysis of Ultrasonic Vocalizations

**Figure 3:**
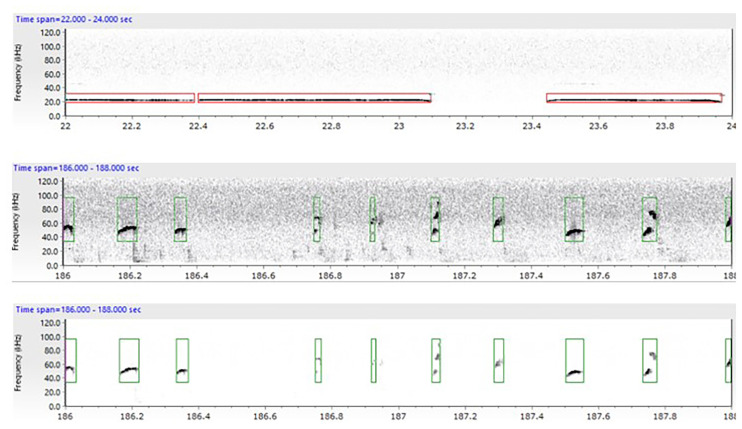
Example spectrogram extracted from the UltraVox XT version 3.2.108 program depicting both 22 kHz (upper image) and 50 kHz calls (lower images). The spectrogram shows the calls emitted in a specific timeframe while being tested in the liberation task. Each spectrogram depicts the communication between the two animals. The red boxes indicate the automatic measurements of calls in the 18–32 kHz frequency range (alarm calls) while the green boxes indicate calls in the 32–96 kHz frequency range (appetitive calls). In the lower panel background noise has been removed.

For the complex housing experiment, USVs of the first (FR1) session in the liberation task were analyzed. Ultrasonic vocalizations were recorded with Avisoft Bioacoustics RECORDER version 4.2.18 (Avisoft Bioacoustics, Berlin, Germany) and analyzed with UltraVox (Noldus Information Technology, Wageningen, the Netherlands). It was not possible to distinguish calls from individual rats, so USVs represent the communication between both rats. Spectrograms were produced from the recordings by fast Fourier transformation (977 Hz frequency resolution, 1.024 ms time resolution, 256 FFT-length, 100% Frame, and 70% time window overlap). These were subsequently scanned using automated parameter measurements for two categories of calls: 22 kHz alarm calls (frequency: 18–32 kHz, min amplitude: 70) and 50 kHz appetitive calls (frequency: 32–96 kHz, min amplitude: 100) according to Wöhr and Schwarting (2013). For both call categories, sounds were labeled as calls if the duration was at least 3 ms. Multiple fragments of calls (or syllables) were labeled as one call if the silence between fragments was less than 3 ms (see Figure 3 for example spectrograms). A lower cut-off-frequency was used to remove all background noise below 18 kHz. The number, duration, and start time of USVs were extracted for further analyses. The reported variable is the percentage of time that rats emit 22 or 50 kHz before and after door opening (calculated as (total duration of 22 kHz or 50 kHz calls (s)/task duration before or after door opening (s)*100%)). Frequencies and average duration of each call type were also examined.

### Statistical Analysis

Statistical analyses were performed using SPSS for windows version 23 (IBM, United States). Results are presented as mean ± SEM. No outlying data points (defined as 3.29 standard deviations below or above the mean) were detected. To compare emergence latency between test rat and partner rat in the boldness test, two way ANOVAs were conducted with role (test- or partner rat) and treatment (no-MD vs. MD; or standard housing vs. complex housing) as between subjects factors. Performance in the progressive ratio task for sucrose was analyzed with independent Student’s *t*-tests. Behavior in the liberation task for pilot experiment A was analyzed using a repeated measures ANOVA with configuration (three different configurations) as within subjects factor, followed by *post hoc* *t*-tests with Bonferroni correction for multiple testing. A paired samples *t*-test was conducted to analyze the two different configurations in pilot B. For the MD and complex housing experiments, (early) life effects were analyzed with independent Student’s *t*-tests for each configuration separately (since not all animals were tested in each configuration) and configuration effects were analyzed with repeated measures ANOVAs. To assess the relationship between motivation to work for a sucrose reward and motivation to liberate a trapped cage mate, Pearson’s correlations were computed in the complex housing experiment.

To analyze the effect of housing on ultrasonic communication before door opening, independent Student’s *t*-tests were performed. To test the effect of door opening (liberation) on USVs, repeated measures ANOVAs were conducted within each call type with door opening (before vs. after) as within subjects factor and housing as between factor, followed by post-hoc *t*-tests with Bonferroni correction for multiple testing if applicable. For a proper comparison of USVs before and after door opening, the pairs in which the test rat did not open the cylinder door(s) within the 10 min of the test (depicted in orange in Figures 6, 7), were not included in this analysis. To compare the number of alarm calls and appetitive calls in the liberation task, repeated measures ANOVAs were conducted with call type (22 kHz vs. 50 kHz calls) and door opening (before vs. after) as within-subjects factor and housing as between-subjects factor. To determine if ultrasonic communication before door opening (liberation) was related to the door opening latency, multiple regression analyses were performed for 22 kHz and 50 kHz calls separately with door opening latency as the dependent variable and USVs and housing conditions as predictor variables. Where appropriate, partial eta squared $\eta_{\text{p}}^{2}$, Hedges’s *g_s_* or *g_av_* are used to present effect sizes according to Lakens (2013).

**Figure 4:**
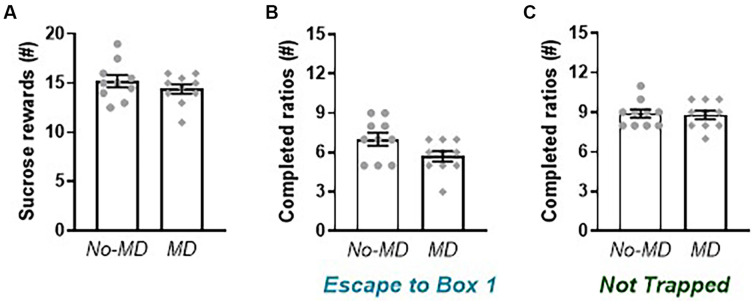
Effect of maternal deprivation on sucrose reward and behavior in the liberation task. **(A)** The number of sucrose rewards earned in a progressive ratio design. **(B)** Motivation to liberate a cage mate (number of completed ratios) in the *Escape to Box 1* configuration in an adapted progressive ratio design. **(C)** Motivation to open the doors in the *Not Trapped* configuration. Data represent individual data points and mean ± SEM, *n* = 10 per experimental group.

**Figure 5:**
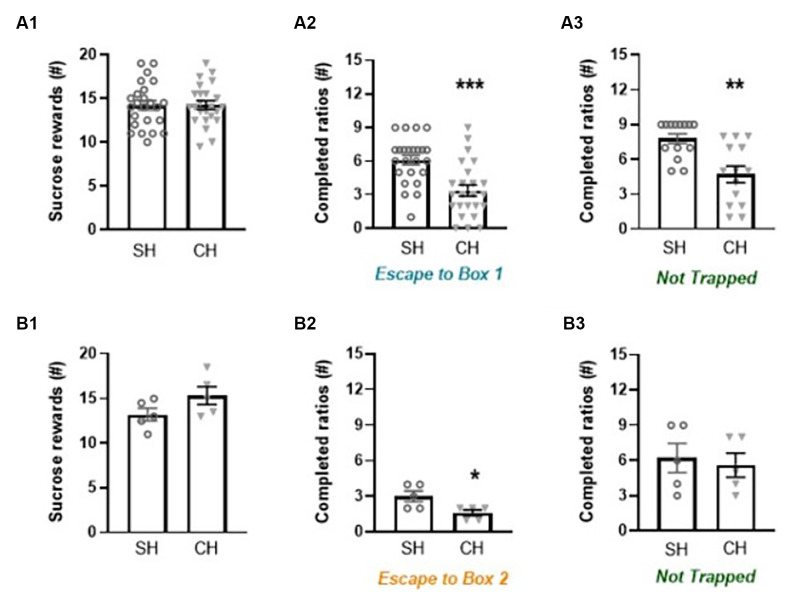
Effect of complex housing on sucrose reward and behavior in the liberation task. The motivation to work for sugar (number of sucrose rewards earned) in a progressive ratio design is depicted in the **(A1)** and **(B1)** graphs. The motivation to liberate a cage mate (number of completed ratios) in an adapted progressive ratio design is depicted in the **(A2)** and **(B2)** graphs. Motivation to open the doors in the *Not Trapped* configuration is depicted in the **(A3)** and **(B3)** graphs. Panel **(A)** shows the main experiment with *Sucrose reward (n = 24 in each exp group)*, *Escape to box 1* (*n = 24 in each exp group*) and *not Trapped* (*n = 14 in each exp group*) configurations, and Panel **(B)** shows preliminary data with *Sucrose reward*, *Escape to box* 2 a*nd not Trapped configurations (n = 5 in each exp group)*. Data represent individual data points and mean ± SEM. *p < 0.05, **p < 0.01, ***p < 0.001.

**Figure 6:**
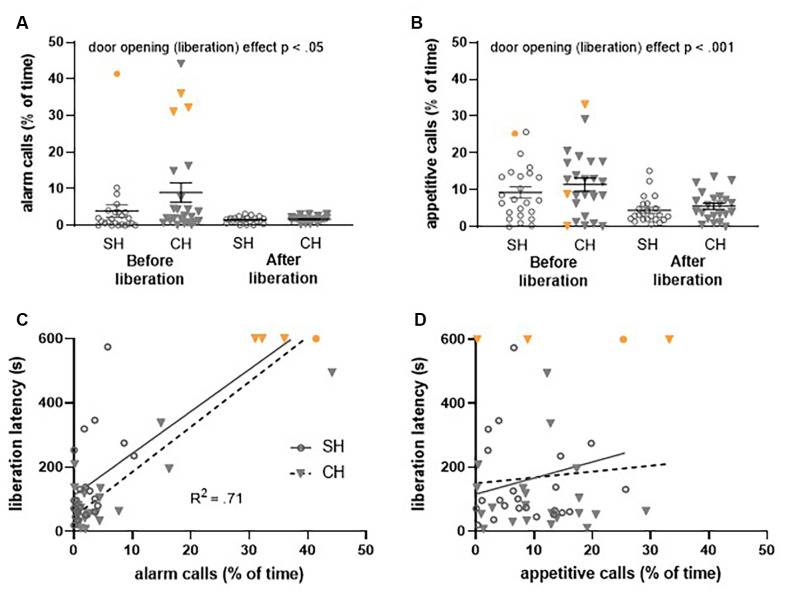
Ultrasonic vocalizations (USVs) recorded during the first (FR1) trial of the *Escape to Box 1* configuration, in the complex housing experiment. Vocalizations are presented as the percentage of time rats emit these USVs. Per experimental group, 24 pairs of rats were included of which four rats (three complex housed and one standard housed rat) did not liberate their cage mates (data points depicted in orange). For a proper comparison of USVs before and after door opening, these data points were not included in the statistical analysis. **(A)** 22 kHz (alarm) calls and **(B)** 50 kHz (appetitive) calls before and after liberation for standard and complex housed rats. **(C)** The relation between 22 kHz vocalizations (before liberation) and liberation latency and **(D)** the relation between 50 kHz vocalizations (before liberation) and liberation latency. SH, standard housed (black line); CH, complex housed (dashed line). Data represent individual data points and mean ± SEM.

**Figure 7:**
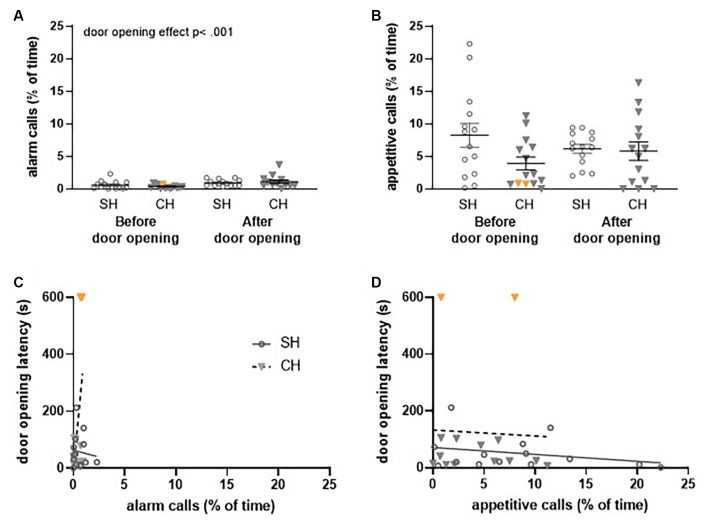
Ultrasonic vocalizations (USVs) recorded during the first (FR1) trial of the *Not Trapped* configuration in the complex housing experiment. Vocalizations are presented as the percentage of time rats emit these USVs. Per experimental group, 14 pairs of rats were included of which two rats (complex housed) did not open the doors (data points depicted in orange). **(A)** 22 kHz (alarm) calls and **(B)** 50 kHz (appetitive) calls before and after door opening for standard and complex housed rats. **(C)** The relation between 22 kHz vocalizations (before door opening) and door opening latency and **(D)** the relation between 50 kHz vocalizations (before door opening) and door opening latency. SH, standard housed (black line); CH, complex housed (dashed line). It should be noted that the number of time animals emitted alarm calls was very low in this configuration. For this reason, the scaling of Figure 7 differs slightly from Figure 6. Data represent individual data points and mean ± SEM.

## Results

### Boldness Measures

The most bold animal of a pair was selected to be the test rat in the liberation task according to Bartal et al. (2011). In both the maternal deprivation and complex housed experiments, test rats and partner rats differed significantly in emergency latency, irrespective of (early life) background (*MD exp*: test/partner role *F*_(1,36)_ = 15.41, *p* < 0.001, *η^2^* = 0.30; role*MD *F*_(1,36)_ = 0.30, *p* = 0.58, *η^2^* = 0.008 and *CH exp*: test/partner role *F*_(1,92)_ = 9.23, *p* < 0.01, *η^2^* = 0.09; role*housing *F*_(1,92)_ = 1.88, *p* = 0.17, *η^2^* = 0.02). Rats selected as test rats had a shorter emergence latency compared to partner rats (*MD exp*: test rats mean: 14.6 s ± 1.5 vs. partner rats mean: 39.6 s ± 6.1, *CH exp*: test rats mean: 17.7 s ± 2.5 vs. partner rats mean: 29.2 s ± 3.6). Maternal deprivation itself did not affect boldness (MD effect: *F*_(1,36)_ = 0.16, *p* = 0.69, *η^2^* = 0.004). Complex housing however did affect boldness (housing effect: *F*_(1,92)_ = 33.39, *p* < 0.001, *η^2^* = 0.29), with a shorter emergence latency for complex housed animals (standard housed mean: 31.1 s ± 3.7 vs. complex housed mean: 13.6 s ± 0.9).

### Task Development

Three pilot experiments (pilots A-C) were conducted to develop the liberation task (for results, see Supplementary Figure 1). Different liberation configurations were tested in a different order in the three pilots (see Figure 1A). The results from the three pilots suggest that testing order is important in this task; specifically in the Trapped condition compared to the freely moving condition. Extensive habituation of the trapped rat to the set-up (if the *Not Trapped* configuration is tested first) might account for this. Following these results, the *Escape conditions (to Box 1 or 2)* were always tested as first configurations in the main experiments, before the *not Trapped* condition.

### Maternal Deprivation Effects on Motivation for Sucrose and Liberation of a Trapped Cage Mate

The motivation to work for a sucrose reward in a progressive ratio design (Figure 4A), was not affected by MD (*t*_(18)_ = 1.02, *p* = 0.32, *g_s_* = 0.44). Rats earned on average around 15 sucrose pellets, which roughly corresponds to 95 lever presses to obtain a sucrose pellet. In the liberation task, MD rats seemed less motivated to liberate a trapped cage mate in the *Escape to Box 1* configuration (Figure 4B), as seen by a lower number of completed ratios, but this effect was (just) not significant, although the effect size was quite large (*t*_(18)_ = 2.05, *p* = 0.06, *g_s_* = 0.88). Rats in the no-MD group completed on average 7 ± 0.5 progressive ratios when liberating a cage mate into box 1, which corresponds to a breakpoint of 15, meaning 15 lever presses are needed to open the door and liberate a cage mate. Rats in the MD group completed on average 5.7 ± 0.4 progressive ratios, lever pressing 9 to 12 times to liberate a cage mate. In the *Not Trapped* configuration (Figure 4C), motivation for door opening was comparable between groups (*t*_(18)_ = 0.22, *p* = 0.83, *g_s_* = 0.09). When tested in this order, rats were more motivated to open the cylinder doors in the *Not Trapped* configuration, compared to the *Escape to Box 1* configuration (Configuration *F*_(1,18)_ = 49.13, *p* < 0.001, $\eta_{\text{p}}^{2}$ = 0.73), irrespective of early life experience (Configuration*MD_(1,18)_ = 2.83, *p* = 0.11, $\eta_{\text{p}}^{2}$ = 0.14). In this condition, rats in both groups completed on average nine progressive ratios, lever pressing on average 25 times to access a non-trapped cage mate.

### Complex Housing Effects on Motivation for Sucrose and Liberation of a Trapped Cage Mate

All animals were tested on motivation to work for sugar. The number of sucrose rewards earned in a progressive ratio design (Figures 5A1,B1) was comparable between standard and complex housed rats, indicating an equal motivation to work for sucrose rewards (1st exp: *t*_(46)_ = −0.29, *p* = 0.97, *g_s_* = 0.00; 2nd exp: *t*_(8)_ = −1.68, *p* = 0.13, *g_s_* = −0.98). When complex housed rats were compared to standard housed rats, they showed a reduced motivation to liberate a trapped cage mate in the *Escape to Box 1* configuration (*t*_(46)_ = 4.13, *p* < 0.001, *g_s_* = 1.15, Figure 5A2). Similarly, motivation for door opening in the *Not Trapped* configuration was reduced in complex housed rats (*t*_(26)_ = 3.76, *p* < 0.01, *g_s_* = 1.4, Figure 5A3). Here, the number of completed ratios did not significantly differ between the Escape to box 1 and Not trapped configurations (Configuration *F*_(1,26)_ = 2.86, *p* = 0.10, $\eta_{\text{p}}^{2}$ = 0.09), in either housing condition (Configuration*Housing *F*_(1,26)_ = 0.05, *p* = 0.83, $\eta_{\text{p}}^{2}$ = 0.00).

The preliminary results in the *Escape to box 2* configuration (Figure 5B2) point to a similar direction, that is, complex compared to standard housed rats showed a reduced motivation to liberate a trapped cage mate (*t*_(8)_ = 2.74, *p* < 0.05, *g_s_* = 1.6, Figure 5B2). This difference was not observed in the *Not Trapped* configuration (*t*_(8)_ = 0.37, *p* = 0.72, *g_s_* = 0.21, Figure 5B3). In this setting, animals were more motivated to lever press for door opening in the *Not Trapped* compared to *Escape to Box 2* configuration (Configuration *F*_(1,8)_ = 14.24, *p* < 0.01, $\eta_{\text{p}}^{2}$ = 0.64), irrespective of housing conditions (Configuration*Housing *F*_(1,8)_ = 0.18, *p* = 0.68, $\eta_{\text{p}}^{2}$ = 0.02).

When comparing the motivation to work for sugar with the motivation to work for door opening in both housing conditions, we found a correlation between completed ratios in sucrose reward and in the *Escape to Box 1* configuration (SH: *r* = 0.73, *p* < 0.001; CH: *r* = 0.44, *p* < 0.05), but not between completed ratios in sucrose reward and the *Not Trapped* configuration (SH: *r* = 0.12, *p* = 0.68; CH: *r* = 0.49, *p* = 0.08).

### Ultrasonic Vocalizations in the Liberation Task

In the complex housing experiment, USVs in the first session (FR1) of both the *Escape to Box 1* and *Not Trapped* configuration were recorded and analyzed.

Housing did not affect the emission of alarm calls before door opening (liberation) in either the *Escape to Box 1* configuration (Figure 6A) or the *Not Trapped* configuration (Figure 7A); the emission of 22 kHz calls was comparable between standard and complex housed rats (*Escape to box 1*: *t*_(46)_ = −1.59, *p* = 0.12, *g_s_* = −0.45 and *Not Trapped*: *t*_(26)_ = 1.24, *p* = 0.22, *g_s_* = 0.47). The same was true for the emission of appetitive calls in both configurations (Figures 6B, 7B); 50 kHz vocalizations before door opening (liberation) were comparable between standard and complex housed rats (*Escape to Box 1*: *t*_(46)_ = −0.87, *p* = 0.38, *g_s_* = −0.25, *Not Trapped*: *t*_(26)_ = 1.68, *p* = 0.10, *g_s_* = 0.62).

As a result of door opening (liberation) in the *Escape to Box 1* configuration, USVs emissions significantly decreased for both call types (Figures 6A,B), irrespective of housing condition (*alarm calls*: door opening *F*_(1,42)_ = 4.73, *p* < 0.05, $\eta_{\text{p}}^{2}$ = 0.10, door opening*housing *F*_(1,42)_ = 2.01, *p* = 0.16, $\eta_{\text{p}}^{2}$ = 0.05; *appetitive calls*: door opening *F*_(1,42)_ = 36.33, *p* < 0.001, $\eta_{\text{p}}^{2}$ = 0.46, door opening*housing* F*_(1, 42)_ = 0.43, *p* = 0.51, $\eta_{\text{p}}^{2}$ = 0.01). The higher amount of USV emission before door opening does not coincide with a higher frequency, but rather a longer duration of calls (see results Supplementary Figure 2). In the *Not Trapped* configuration (Figure 7A), the emission of alarm calls significantly increased after door opening, irrespective of housing condition (*F*_(1,24)_ = 11.97, *p* < 0.01, $\eta_{\text{p}}^{2}$ = 0.33, door opening* housing *F*_(1,24)_ = 1.84, *p* = 0.19, $\eta_{\text{p}}^{2}$ = 0.07), although both before and after door opening levels were very low. For the appetitive calls, a significant interaction was found between door opening and housing (*F*_(1,24)_ = 5.87, *p* < 0.05, $\eta_{\text{p}}^{2}$ = 0.19), with emission of vocalizations seemingly decreasing after door opening for the standard housed rats and increasing for the complex housed ones. Post-hoc analysis however, did not show statistically significant difference between before and after door opening in either group (SH: *t*_(13)_ = 1.6, *p* = 0.13, *g_s_* = 0.41, CH: *t*_(11)_ = − 1.87, *p* = 0.09, *g_s_* = 0.53).

In general, in both the *Escape to Box 1* and the *Not Trapped* configuration, rats spent more time emitting appetitive calls than alarm calls, both before and after door opening and irrespective of housing conditions (*Escape to box 1*: call type *F*_(1,42)_ = 33.73, *p* < 0.001, $\eta_{\text{p}}^{2}$ = 0.44, call type*door opening *F*_(1,42)_ = 1.97, *p* = 0.05, $\eta_{\text{p}}^{2}$ = 0.09, call type*housing *F*_(1,42)_ = 0.007, *p* = 0.93, $\eta_{\text{p}}^{2}$ = 0.00; *Not Trapped*: call type *F*_(1,24)_ = 46.02, *p* < 0.001, $\eta_{\text{p}}^{2}$ = 0.66, call type*door opening *F*_(1,24)_ = 0.25, *p* = 0.62, $\eta_{\text{p}}^{2}$ = 0.01, call type*housing *F*_(1,24)_ = 0.60, *p* = 0.45, $\eta_{\text{p}}^{2}$ = 0.02).

The emission of alarm calls before liberation and the influence of housing condition could explain a significant amount of the variance observed in the latency to liberate in the *Escape to Box 1* configuration (Figure 6C, *F*_(2,45)_ = 54.59, *p* < 0.001, *R^2^* = 0.71); the analysis shows that higher emission rates of alarm calls were observed when liberation of the cage mate took longer (*Beta* = 0.86, *t*_(47)_ = 10.45, *p* < 0.001) and complex housed animals tended to have higher liberation latencies (Beta = −0.18, *t*_(47)_ = −2.14, *p* < 0.05). This was not observed for the appetitive calls (Figure 6D, *F*_(2,45)_ = 0.48, *p* = 0.62, *R^2^* = 0.02). Thus, in the *Not Trapped* configuration, the emission of alarm calls before door opening and housing condition did not predict the variance observed in the latency to door opening (Figure 7C, *F*_(2,25)_ = 1.06, *p* = 0.36, *R^2^* = 0.08), and neither did the appetitive calls (Figure 7D, *F*_(2,25)_ = 0.79, *p* = 0.46, *R^2^* = 0.06).

## Discussion

In the present study, we investigated the effect of (early) life challenges on motivation for pro-social behavior. To this end, we developed an automated, operant pro-social liberation task for rats aimed at measuring motivation to liberate a trapped conspecific. Rats learned to press a lever that opened the door of a cylinder in which a partner rat was trapped. Manipulation of the early postnatal environment by 24 h MD at postnatal day 3 did not affect behavior in this task, regardless of whether the cage mate was trapped or freely moving in a separate compartment. Rearing rats in complex housing from postnatal day 26 onwards did affect behavior; compared to standard housed rats, complex housed rats displayed a similar motivation to press a lever for sucrose, yet a reduced motivation to liberate a trapped cage mate as well as a reduced motivation to gain access to a non-trapped rat. During the first test session with either a trapped or not trapped cage mate, emission of both 22 kHz and 50 kHz calls was comparable between standard housed and complex housed rats, suggesting that USVs are not necessarily linked to the reduced motivation of door opening in the complex housed animals. With this novel operant task, we showed that rats are motivated to liberate a trapped cage mate, even when liberation does not lead to social contact. Moreover, we showed that rats are more motivated to lever press and gain access to the cage mate when the cage mate is not trapped but freely moving in a separate compartment. This suggests that rats are affected by the emotional state of the partner and adapt their behavior depending on whether or not the partner is trapped, though in this case trapping of the partner *reduced* motivation to act. In support of this finding, analysis of USVs revealed that there was a positive correlation between the amount of 22 kHz alarm calls (and not 50 kHz appetitive calls) emitted before liberation and the latency to door opening, suggesting that increased levels of distress were linked to a decreased motivation for door opening.

### Challenging Early Life Conditions

Two separate experimental conditions were introduced to study how the (early) life environment affects the development of pro-social behavior. First, the early postnatal environment was manipulated by depriving pups on postnatal day 3 from maternal care for 24 h. In rodents, stressful experiences in the early postnatal period, including maternal deprivation, can alter stress reactivity and cause deficits in cognitive and emotional behavior (Lupien et al., 2009; Krugers and Joëls, 2014; Marco et al., 2015; Van Bodegom et al., 2017; Bonapersona et al., 2019). Regarding social behavior, early life stress studies have found limited effects on social interest, but mostly a decrease in social discrimination and a decrease in (or no effect on) social interaction (van der Veen et al., 2020). Whether or not an animal acts to help a distressed conspecific is a complex context-dependent process and it is likely that individual behavioral characteristics such as anxiousness and ability to self-regulate emotions affect the response to distressed others.

To our knowledge, the current research is the first to study the effects of early life stress on pro-social behavior. Compared to non-deprived controls, MD animals tended to show lower levels of pro-social activity in the Trapped configuration, with quite high effect size, but not reaching statistical significance, most likely due to the relatively small sample sizes. Performance in the Not Trapped configuration was very similar in MD rats and non-deprived controls. As part of lever acquisition training, rats also conducted a progressive ratio for sucrose which allowed us to compare motivation to work for a food reward with motivation to work for a social reward. MD animals were equally motivated to work for a sucrose reward as non-deprived controls. Together with performance in the liberation task, it seems that reward sensitivity, both for food and a social reward, is not strongly affected by MD, at least not in the current experimental design.

A second environmental manipulation concerned complex housing from early adolescence onwards, starting on postnatal day 26 and lasting throughout testing. Complex housed rats were less motivated to liberate a trapped cage mate, as reflected by a significant reduction in the number of completed ratios compared to standard housed controls in both the *Escape to box 1* (towards) and *Escape to box 2* (away from) configurations. This reduction in motivation is probably not explained by increased emotional contagion or reduced behavioral self-regulation, because motivation in the *Not Trapped* configuration was also reduced. Even though there are indications of increased corticosterone concentrations in both rats (Moncek et al., 2004) and mice (Haemisch et al., 1994; Marashi et al., 2003) kept in enriched environments, previous experiments have shown that complex housed rats quickly adjust to novel environments and show a reduced behavioral response to mild novelty stress (Zimmermann et al., 2001; van der Veen et al., 2015; Kentrop et al., 2018). We hypothesized that complex housed partners might experience less distress from being trapped in the cylinder than standard housed partners, which, as a result, could lower the urgency for complex housed rats to liberate their partner. However, the emission of 22 kHz alarm calls before liberation was equal in complex and standard housed groups, suggesting that at least in the first test session partners from both groups experienced equal distress from being trapped. Interestingly, the emission of 50 kHz calls was also higher before than after the door opening. These 50 kHz calls however were not correlated with liberation latency. This pattern of results suggests more communication in general before liberation. It could be hypothesized that reduced motivation to act in both configurations might reflect an overall reduced motivation to work for a social reward. Moreover, this decrease in motivation appeared to be specific for social rewards, as the motivation to work for a sucrose reward was comparable for complex and standard housed rats. These results seem to be in accordance with recent findings of our lab where complex housed rats were tested in a pro-social two-choice task. In this task, rats were given the choice between lever pressing to obtain a sucrose reward for themselves or for themselves and their partner. Standard housed rats on average preferred the pro-social option, while complex housed rats did not (Kentrop et al., 2020). We have also previously shown that testing complex housed rats shortly after taking them from the (complex) home cage reveals a strong reduction in social interest compared to standard housed animals (Kentrop et al., 2018). This reduced social interest -as a result of living with 10 conspecifics- could also affect the reward experienced by liberating or gaining access to another rat and lead to less pro-social behavior. Future experiments should look into possible neurobiological mechanisms underlying the behavior observed.

### Task Development

Our paradigm was inspired by the seminal work of Bartal and colleagues (Bartal et al., 2011, 2014, 2016). In these studies, rats learned over time to manually open a cylinder that contains a trapped conspecific. As the requirements for door opening do not change over the course of testing, an increase in the percentage of door openings and decrease in door opening latencies over sessions demonstrated that liberation of the cage mate is reinforcing and stimulates repeated liberation. A similar study with similar findings was performed by Sato et al. (2015), in which the partner was trapped in a pool of water instead of a cylinder.

In our set-up, the requirements for door opening increased over sessions—to probe motivation—until the rats ceased to meet the requirements and the experiment ended. Because we measured motivation in a progressive ratio design, this allowed us to compare motivation to work for a social reward with motivation to work for a food reward. While most prior studies find social and food reinforcers to be roughly equal (Evans et al., 1994; Bartal et al., 2011; Sato et al., 2015), in our study the motivation (amount of work provided) for a sucrose reward was higher compared to the motivation for social contact in the non-trapped condition or in either of the liberation conditions for that matter. Hiura et al. (2018) found a similar result in their study regarding the social release paradigm. It is important, however, to take into consideration two facts. Firstly, a straight comparison of the performance of the rats in the two tasks is not possible as they were performed in different setups with different protocols. Moreover, in our experiments rats were slightly food-deprived before being tested for sucrose reward while they were not socially deprived before being tested in the liberation task. We deliberately did not deprive the rats from social contact before testing, since the debate surrounding the liberation task is the inability to disentangle whether lever pressing is (partly) motivated by the desire for social contact or is based on the desire to help the trapped rat (Schwartz et al., 2017; Hachiga et al., 2018). Similar to the current study, Bartal and colleagues separated the desire for social contact from ending the distress of a conspecific in a control experiment in which the rat was separated into a second compartment and showed that the desire for social contact was not the main force driving behavior. The current data set complements these earlier studies, by replicating the finding that rats are indeed willing to liberate a trapped conspecific repeatedly and with increased cost, although the first results do suggest that liberation in a separate compartment decreases the motivation to do so. By introducing the non-trapped configuration, we showed that the motivation to gain access to a freely moving cage mate is higher than the motivation to liberate a trapped cage mate with subsequent social contact. This suggests that rats are affected by the emotional state of the partner and adapt their behavior depending on whether or not the partner is trapped, though in this case trapping of the partner *reduced* motivation to act. In support of this finding, analysis of USVs revealed that there was a positive correlation between the amount of 22 kHz alarm calls (and not 50 kHz appetitive calls) emitted before liberation and the latency to door opening, suggesting that increased levels of distress were linked to a decreased motivation for door opening.

If through emotional contagion, rats are negatively affected by the distressed state of the trapped partner, rats are expected to downregulate their own distress by liberating the partner. When we started testing, we placed the partners in a much smaller cylinder in which animals could not move or turn and showed clear physical signs of distress (such as actively struggling, excretion of feces, and urinating). In this setting, test rats did not press the lever at all, but rather avoided the cylinder. This avoidance of stressed conspecifics by male rats has also been reported by others (Rogers-Carter et al., 2018). On the other hand, Bartal et al. (2016) indicated that rats that were less stressed were also less inclined to show prosocial behavior. In the current setting, animals did press the lever to liberate a cage mate, but the number of completed ratios mostly remained lower than in the *Not Trapped* configuration. Taken together, data from the current and other studies show that there is a delicate window in which vicarious distress induces other rats to act pro-socially. In this study, we only looked into the behavior of male rats as a first step to establish our automated operant version of the liberation task. Due to the fact that behavioral testing for this task is particularly long and time consuming, it was not possible, unfortunately, to simultaneously test female animals. We certainly consider testing females an important next step.

Both 22 kHz alarm calls and 50 kHz appetitive calls were recorded during the first test session (FR1) of both the Trapped and Not Trapped configurations. If the liberation of a trapped rat is motivated by a shared affective state *via* emotional contagion, then ultrasonic communication is a prime candidate for the transfer of emotions between individuals (Brudzynski, 2013). In the Trapped configuration, the amount (depicted in the percentage of time that calls were emitted) of both 22 kHz alarm and 50 kHz appetitive calls decreased after door opening, which was not observed in the Not Trapped configuration. This indicates that USVs may play a larger role in communication when the partner is trapped and subsequently liberated than in non-trapped conditions. It is to be noted that USVs were recorded per pair of animals and it was not possible to identify the source of the alarm calls (test rat or partner). Ultrasonic communication before liberation also correlated with the behavior of the test rat; i.e., higher emission rates of 22 kHz alarm calls coincided with longer door opening latencies in the trapped condition. In light of our previous observations, we speculate that higher emission rates of 22 kHz calls delay door opening latency, but it might also be true that longer door opening latencies increase 22 kHz vocalization. Nonetheless, the positive correlation between alarm calls and liberation latency suggests that ultrasonic communication might contribute to the process of emotional contagion and that increased levels of distress might reduce active helping behavior. These preliminary results clearly await more in-depth investigation.

### Conclusion

In conclusion, we developed and tested an adapted version of a behavioral task that measures pro-social behavior in rats. We show suggestive evidence that rats are sensitive to the distressed state of trapped cage mates and attempt to liberate them; and that this might be affected by challenging conditions during (early) life. Moreover, the task demonstrated to be sufficiently sensitive to measure motivation for door opening in different contexts, which allows for future experiments that examine contextual prerequisites needed for individuals to behave pro-socially.

## Data Availability Statement

The raw data supporting the conclusions of this article will be made available by the authors, without undue reservation.

## Ethics Statement

The animal study was reviewed and approved by Central Authority for Scientific Procedures on Animals in the Netherlands.

## Author Contributions

JK, RV, AK, MJ, MI, and MB-K contributed to the conception and design of the study. JK, AK, and RV were responsible for the execution of the experiments, the data collection, the statistical analyses, and the composition of the first draft of the manuscript. CH and EG were involved in the execution of the experiment and the collection of the data. All authors contributed to the article and approved the submitted version.

## Conflict of Interest

The authors declare that the research was conducted in the absence of any commercial or financial relationships that could be construed as a potential conflict of interest.

## Publisher’s Note

All claims expressed in this article are solely those of the authors and do not necessarily represent those of their affiliated organizations, or those of the publisher, the editors and the reviewers. Any product that may be evaluated in this article, or claim that may be made by its manufacturer, is not guaranteed or endorsed by the publisher.
