# Supplementary material for: Complex Housing, but Not Maternal Deprivation Affects Motivation to Liberate a Trapped Cage-Mate in an Operant Rat Task
Source: Front Behav Neurosci. 2021 Aug 26;15:698501. doi: 10.3389/fnbeh.2021.698501 (PMC8427758; doi:10.3389/fnbeh.2021.698501)
Supplement: Supplementary file 1 [file Data_Sheet_1.pdf]

Supplementary material

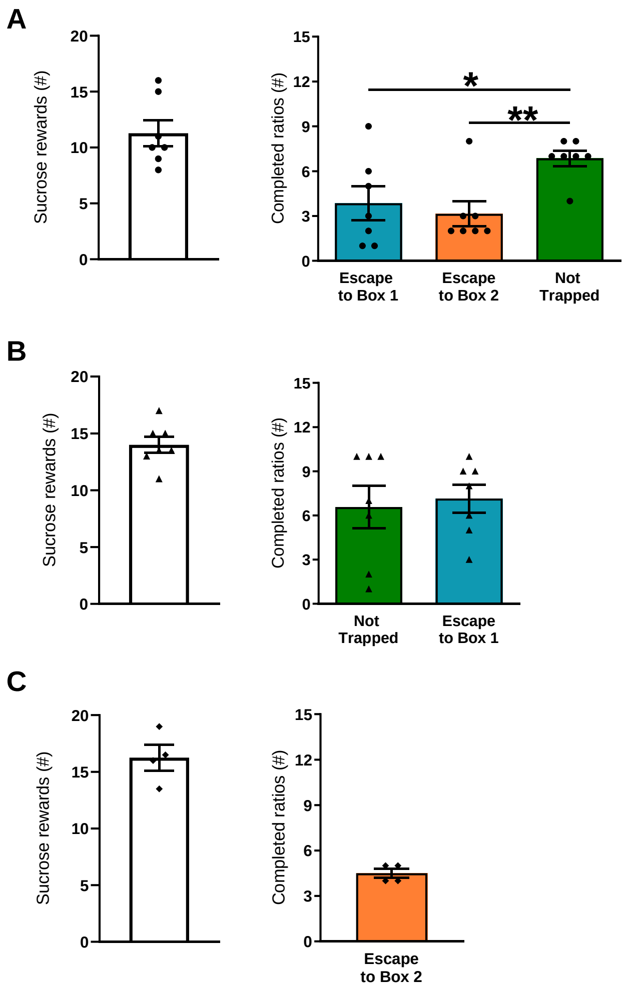

Figure S1: Pilot experiments with the liberation task. The number of sucrose rewards earned in a progressive ratio design is plotted in the graphs on the left (white bars). The motivation to liberate a cage mate (number of completed ratios) in an adapted progressive ratio design is plotted in the graphs on the right (blue, orange and green bars). (A) Results for pilot A, where the Escape to box 1 configuration was tested first, followed by Escape to box 2 and the Not Trapped configuration (*n* = 7). Motivation for door opening differed between configurations (Configuration ($F(2,12) = 11.54$, $p < .01$, $\eta p^2 = .66$). (B) Results for pilot B, where the Not Trapped configuration was tested first, followed by Escape to Box 1 (*n* = 7). No difference was found between the two configurations ($t(6) = -0.76$, $p = .48$, $g_{av} = 0.15$) (C) Results for pilot C, where Escape to Box 2 was tested as a first configuration (*n* = 4). These results were comparable to the Escape to box 1 condition in pilot A. Data represent individual data points and mean ± SEM. *p < .05, **p < .01.

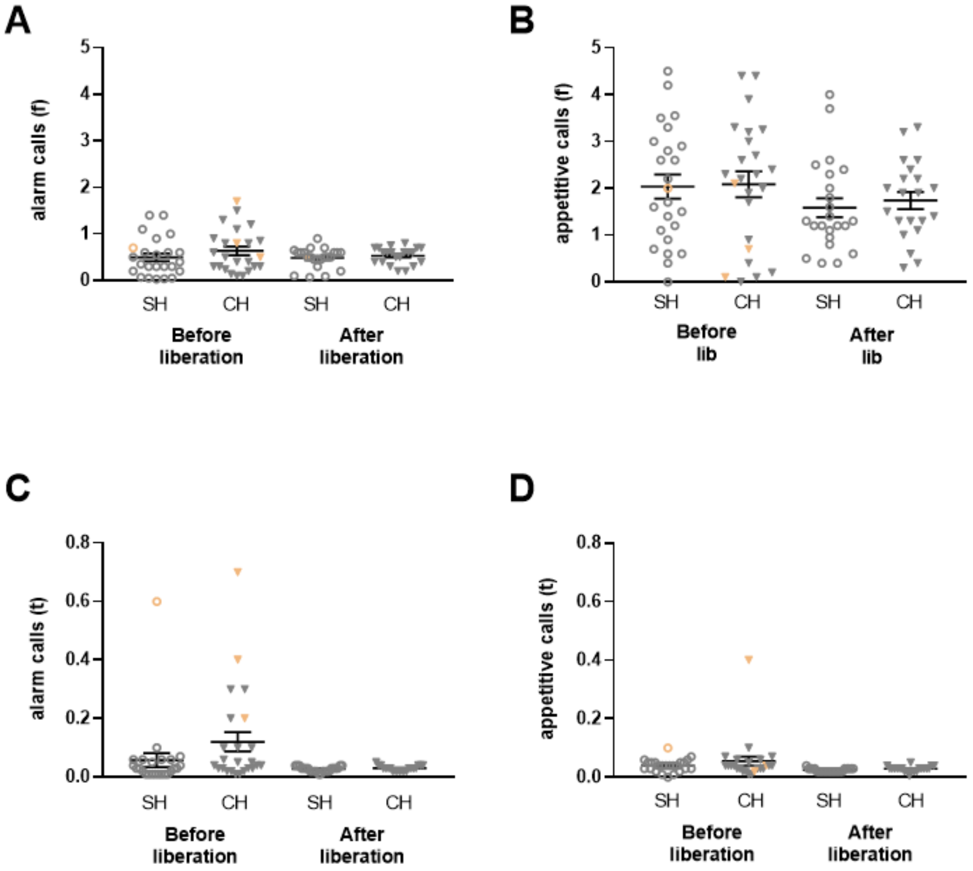

Figure S2: Frequency and duration of USVs in the Escape to Box 1 condition. A) the frequency (f) of the alarm calls emitted by the rats during the Escape to Box 1 condition. B) the frequency (f) of the appetitive calls emitted by the rats during the Escape to Box 1 condition. C) the duration (t) of the alarm calls emitted during the Escape to Box 1 condition, D) the duration (t) of the appetitive calls emitted during the Escape to Box 1 condition.
